# Health Literacy in the Population of Diabetic Patients in Iran: A Systematic Review and Meta-Analysis

**Published:** 2020-04

**Authors:** Marjan MOMENI, Majid MIRMOHAMMADKHANI, Abbas ZIARI

**Affiliations:** 1. Department of Health Information Technology, School of Allied Medical Sciences, Semnan University of Medical Sciences, Semnan, Iran; 2. Social Determinants of Health Research Center, Semnan University of Medical Sciences, Semnan, Iran

**Keywords:** Health literacy, Diabetic, Systematic review, Meta-analysis, Iran

## Abstract

**Background:**

The present systematic review and meta-analysis were conducted to find the degree by which the Iranian diabetic patients were informed about their disease in term of health literacy.

**Methods:**

The search was carried out in databases including the PubMed, MEDLINE (PubMed), Scopus, Embase, Cochrane Library, ProQuest, Web of Science, Science Direct and Wiley and also domestic databases including the Iranian Academic Center for Education Culture and Research (sid.ir), the Iranian Research Institute for Information Science and Technology (IranDoc.ac.ir), Barakat Knowledge Network System (barakatkns.com), the national publications database (magiran.com) and also Google Scholar and Elmnet search engines. All the original studies published by Oct 20, 2018, in Persian or English, to assess the health literacy of adults with diabetes were included in the study. Ultimately, 17 articles were included in the meta-analysis. The meta-analysis was carried out using the fixed-effects method using Stata-11.2.

**Results:**

The pooled mean score of health literacy was 56.65 out of score of 100, and its 95% CI was estimated as 49.85 to 63.45. No significant heterogeneity could be reported for the articles included in the meta-analysis (I-squared=21.3%, *P*=0.177). The pooled mean scores with the CI of 95% by gender based on the study population in women and men were estimated as 47.97and 50.06 respectively.

**Conclusion:**

Health literacy is not high in Iranian diabetic patients and is rather inadequate in most of them in both genders. Diabetic women have somewhat lower health literacy compared to diabetic men.

## Introduction

Diabetes has always been of interest in medical research as a chronic disease and a major health problem (1–3). Some studies have addressed the costs of this disease (4) while others have focused on its complications (5, 6). The number of diabetics is estimated to reach 300 million by 2025 (7). According to WHO, the prevalence of diabetes in Iran in men, women and in general population are 9.6%, 11.1% and 10.3% respectively (8). A greater attention has therefore been paid in recent years to the personal and family aspects of the prevention of this disease (9, 10). In Iran, too, this disease has been investigated in various studies from different perspectives (11, 12). One of the factors affecting the prevention and control of diabetes is having adequate knowledge about the disease, the factors affecting its incidence and the methods of its prevention. Health literacy is one of the factors affecting this knowledge (13).

Many studies have thus been conducted on health literacy and diabetes (14–16). The relationship between health-promoting behaviors and health literacy was investigated in diabetic patients and revealed a significant relationship between health literacy and all the dimensions of health-promoting behaviors in these patients (17). The relationship between health literacy and diabetes control was studied and argued that inadequate health literacy has a significant relationship with poor glycemic control (18). Functional health literacy can play a major role in diabetic patients’ adherence to nutritional recommendations (19). Diabetic patients with poor health literacy have difficulty understanding their medication instructions (16), and this deficiency challenges the control of the disease and its consequences. Health literacy has been defined as people’s capacity to acquire, interpret and understand health information and services, which are essential to proper decision-making (20).

WHO reported health literacy as one of the biggest determinants of health and has recommended all countries throughout the world to establish an association consisting of all those affected by the disease to monitor and coordinate strategic activities for the promotion of health literacy (21). Patients with poor health literacy are hospitalized more frequently and for longer periods compared to those with adequate health literacy. The lack of health literacy is correlated with poor quality of care and imposes an additional burden on health resources (22). People with inadequate health literacy enjoy less health, have false understandings of health information, often wait for longer periods and only seek medical help when their problem has become critical (23). Inadequate health literacy is associated with the inappropriate use of medications, non-adherence to doctors’ instructions and the lack of well-being. The assessment of health literacy is therefore necessary for reducing the likelihood of risks due to its inadequacy (24).

Various studies in Iran have also investigated the relationship between health literacy and diabetes (25, 26). A significant relationship was found between health literacy and general health in patients with type-II diabetes (27, 28). A relationship found between health literacy and self-care in diabetic patients (26, 29). Other studies have confirmed the relationship of health literacy with self-efficacy (30), quality of life (31), adherence to treatment regimen (32) and diabetes prevention (33).

Meanwhile, some studies have pointed to the inadequacy of health literacy among diabetic patients in Iran (25, 34), which shows the need for greater attention to the health literacy of diabetic patients. However, these studies have been conducted on particular groups of the population rather the entire nation.

Health literacy is very important in the prevention and control of diabetes, and the assessment of health literacy in diabetic patients seems necessary for health and medical planning and treatment. Nonetheless, no studies have yet been independently conducted to provide an accurate summary of the level of health literacy in diabetic patients in Iran, and the only study on health literacy in Iran (35), has determined the level of health literacy in different patients without specifying their disease type. Given the significance of diabetes complications and the many factors associated with health literacy in diabetic patients, the assessment of specific health literacy in diabetic patients appears essential.

Since many different factors are associated with the health literacy of diabetic patients, which can be widely different from those in patients with other diseases and the general public, the present systematic review meta-analysis was conducted to determine the level of health literacy in patients with type 2 diabetes in Iran.

## Methods

The proposal for this research was first assessed and approved by the University Research Council (Code: A-10-230-17), and the approval of the University Ethics Committee was then obtained (IR.SEMUMS.REC.1397.045). Next, the preliminary stage of the search was carried out and based on the results obtained, the protocol of the systematic review was drafted and uploaded to PROSPERO website. Once the protocol code was issued by PROSPERO (CRD42018098934), the systematic search was carried out as follows:

### Data Sources and Search strategy

To access the studies conducted on health literacy in diabetic patients in Iran, a search was carried out in databases including the PubMed, MEDLINE (PubMed), Scopus, Embase, Cochrane Library, ProQuest, Web of Science, Science Direct and also domestic databases including the Iranian Academic Center for Education Culture and Research (sid.ir), the Iranian Research Institute for Information Science and Technology (IranDoc.ac.ir), the national publications database (magiran.com) and also Google Scholar and Elmnet search engines. Articles in both Persian and English were retrieved using keywords including “health literacy”, “HL”, “Diabetes”, “Diabetes Mellitus”, “Diabet^*^” and ‘Iran’. The keywords were used both with and without quotation marks and also with wildcards (^*^) and were combined using the Boolean functions ‘AND’ and ‘OR’. A subject search was also carried out using Medical Subject Headings (MeSH) through Medline (PubMed) database. To access all the relevant articles in gray literature, the core journals and eligible article resources were reviewed. To access the latest published studies, an alert system was activated on a number of databases, including PubMed and Scopus, so that any new articles published during the study could also be reviewed. In this study, the search strategy and the screening and selection of the data were based on the Preferred Reporting Items for Systematic Reviews and Meta-analyses (PRISMA) guidelines.

### Selection of studies and Eligibility Criteria (Inclusion & Exclusion criteria)

Since the purpose of this review study was to determine the level of health literacy in adults with diabetes in Iran, no limitations were imposed on the type of study included, and all the original studies published by Oct 20, 2018, in scientific journals, in Persian or English, to assess the health literacy of adults with diabetes, using Test of Functional Health Literacy in Adults (TOFHLA), Health Literacy for Iranian Adults(HELIA) and Short Test of Functional Health Literacy in Adults (S-TOFHLA) were included in the study without any time constraints. The first stage consisted of article search and retrieval. In the next stage, after the elimination of the repeated articles, the titles and abstracts of the retrieved articles were independently assessed by two project collaborators to identify the eligible articles. The assessing team consisted of an epidemiologist, a community medicine specialist and a knowledge and information science PhD. Disagreements about the inclusion of the articles were resolved through discussion and debate to avoid the risk of bias, and in some cases, a third person also made comments. The full text of the eligible articles was then retrieved. The studies that had not numerically specified the level of health literacy in adult diabetic patients were excluded. Duplicated results, irrelevant results, conference abstracts, proceeding papers, books and editorial letters were excluded.

### Study Quality assessment

The full text of the eligible articles was assessed by two independent project collaborators. The two researchers also assessed the quality of the articles independently based on CASP tools for evaluation of cross-sectional studies. Articles answered “yes” by both of two authors in the first two questions and got at least five” yes “from 8 other questions, were selected. The strengths and weaknesses of each article were assessed and noted. To minimize the risk of bias, a comprehensive search was carried out in various local and international databases.

### Data extraction

Data were extracted from the eligible articles entered into the study and included the first author’s name, publication year, city studied, type of questionnaires, gender, habitation, sample size and the mean score of health literacy. For the meta-analysis, the mean and standard deviation values were also extracted. Excel was used for data input and notifying other research team members.

**Fig. 1:**
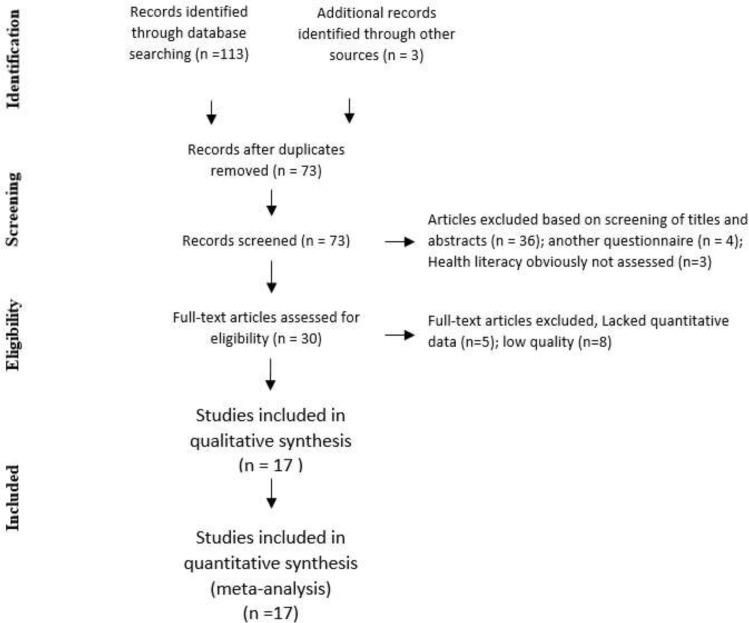
PRISMA Flow Diagram for document selecting

Figure 1 presents the process of article selection for inclusion in the meta-analysis based on the PRISMA Flow Diagram.

Ultimately, 17 articles published between 2012 and 2018 (Table 1) were included in the meta-analysis.

**Table 1:** Basic characteristics of the included studies in the meta-analysis on the health literacy of diabetic population in Iran

***ID***	***First author***	***Gender***	***Year***	***City***	***Habitation***	***Tool***	***Sample size***
1	Tol	female	2012	Tehran	urban	S-TOFHLA	160
2	Khosravi	both	2013	Shiraz	urban	TOFHLA	343
3	Kooshyar	both	2014	Mashhad	urban	S-TOFHLA	300
4	Rafiezadeh Gharrehtapeh	both	2015	Gorgan	urban	HELIA	100
5-a	Mohammadi	female	2015	Tehran	urban	TOFHLA	251
5-b	Mohammadi	male	2015	Tehran	urban	TOFHLA	156
6	Maleki	both	2016	Zahedan	urban	TOFHLA	182
7	Seyedoshohadaee	both	2016	Tehran	urban	TOFHLA	200
8-a	Rezaee Esfahrood	female	2016	Yazd	urban	TOFHLA	72
8-b	Rezaee Esfahrood	male	2016	Yazd	urban	TOFHLA	360
9-a	Moeini	female	2016	Tuyserkan	urban	TOFHLA	65
9-b	Moeini	male	2016	Tuyserkan	urban	TOFHLA	66
10-a	Charoghchian Khorasani	female	2017	Chenaran	urban	TOFHLA	162
10-b	Charoghchian Khorasani	male	2017	Chenaran	urban	TOFHLA	162
11	Arbabi	both	2017	Zabol	urban	TOFHLA	150
12-a	Salimi	female	2017	Shahroud	urban	TOFHLA	277
12-b	Salimi	male	2017	Shahroud	urban	TOFHLA	173
13	Borji	both	2017	Ilam	urban	TOFHLA	250
14	Chahardah-Cherik	both	2017	Ahvaz	urban	TOFHLA	175
15	AbbasZadeh Bazzi	both	2018	Zabol	urban	TOFHLA	150
16-a	Fadaiyan Arani	female	2018	Aran-vabidgol	rural	HELIA	80
16-b	Fadaiyan Arani	male	2018	Aran-vabidgol	rural	HELIA	40
17	Mehrtak	both	2018	Ardabil	urban	S-TOFHLA	241

### Data analysis

The meta-analysis was performed in Stata-11.2 (Copyright 1985–2009) according to the mean command. Publication bias was assessed using the funnel plot drawn based on the data. Heterogeneity was assessed using the I-squared index (values>50 were considered heterogeneous). Data obtained from the female and male populations were separately included in the main meta-analysis process and the analysis of the subgroups, if available. The meta-analysis was carried out using the random-effects method, and a Forrest plot was drawn for each analysis. To pool the data obtained by different tools, the final score of each study was recalculated in the 0–100 range and included in the meta-analysis.

## Results

Data pertaining to the sample population of 4115 assessed in these studies were analyzed. The smallest sample size belonged to Rafiezadeh et al (30) study (n=100) and the largest to Salimi et al (33) study (n=450). To evaluate the level of health literacy, two studies had used the STOFHLA, two had used HELIA and the rest had used TOFHLA. Based on the methodology stated in the articles, the health literacy data were assessed by gender in six of the articles, in an all-female population in one article and irrespective of gender in the rest of the articles. Table 1 presents the data pertaining to the studies included in the meta-analysis.

**Fig. 2:**
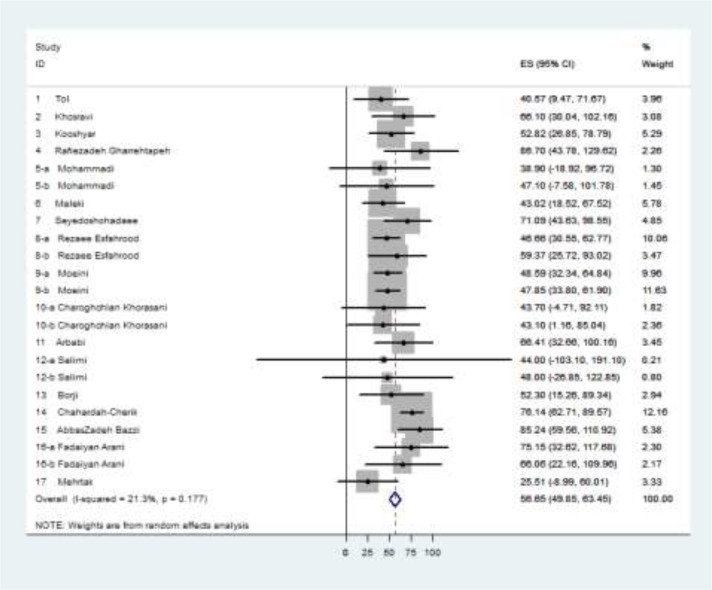
Forest plot for individual and pooled estimated (ES) mean scores of health literacy in the population of diabetic patients in Iran

Figure 2 presents the Forest plot and the mean health literacy scores in each study and also the pooled mean score with the CI of 95% in the population of diabetic patients in Iran. The pooled mean score of health literacy was 56.65 out of a total score of 100, and its 95% CI was estimated as 49.85 to 63.45. No significant heterogeneity could be reported for the articles included in the meta-analysis (I-squared=21.3%, *P*=0.177).

**Fig. 3:**
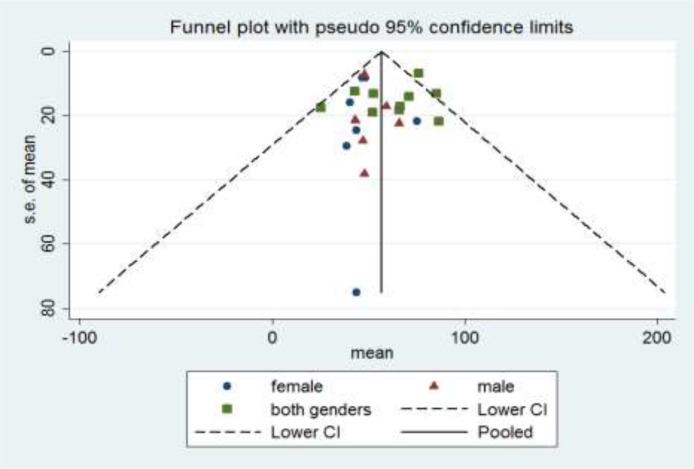
Funnel plot for the graphical assessment of publication bias in the meta-analysis by gender

Figure 3 presents the funnel plot for the graphical assessment of publication bias in the meta-analysis.

**Fig. 4:**
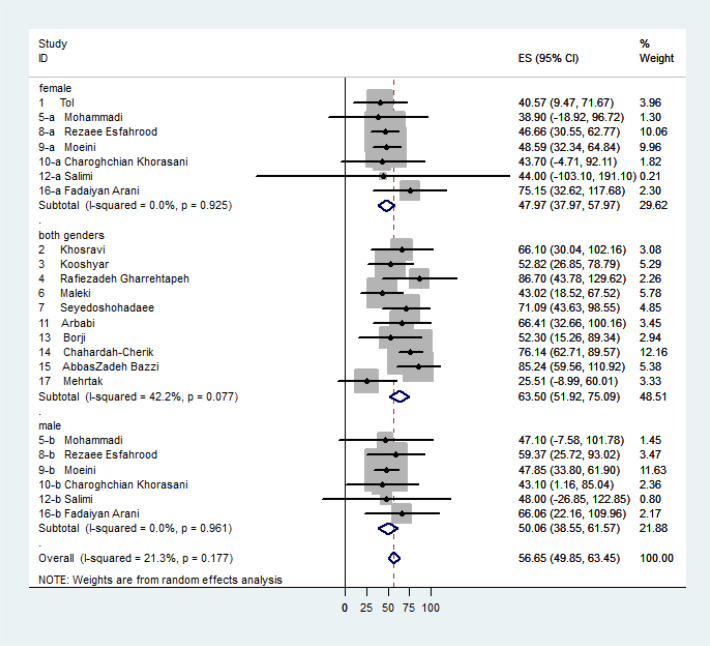
Forest plot for individual and pooled estimated (ES) mean scores of health literacy in the population of diabetic patients in Iran by gender

Figure 4 presents the Forest plot and the mean health literacy scores in each study and also the pooled mean scores with the CI of 95% by gender based on the study population in three subgroups, i.e. women, men and mixed gender. These scores were estimated as 47.97, 50.06 and 63.50 respectively.

## Discussion

The present study is the first systematic review and meta-analysis of the level of health literacy in diabetic patients in Iran. The results of the review of 17 articles revealed a mean health literacy score of 56.65 (out of 100) in Iranian diabetic patients, and since most of the studies had used a rating system of ‘adequate’, ‘borderline’ and ‘inadequate’ to rate the level of health literacy in their subjects (Patients are categorized as having adequate, marginal, and inadequate health literacy by two-point scores of 59 and 74) (25), the majority of Iranian diabetic patients can be said to have lacked an adequate health literacy.

This result agrees with the findings of a study conducted in Pakistan (a neighboring country of Iran), in which health literacy was inadequate in 67.15% of the patients, which means that the majority of the diabetes patients had poor health literacy (36). In a study in Kuwait (another neighboring country of Iran), also showed a generally inadequate health literacy in most diabetic patients (37).

In the US, to compare health literacy in older diabetic patients and older healthy adults showed that poor health literacy was higher in older male diabetic patients (10.1%) compared to healthy older men (9.3%), which also applied to women (14.7% vs. 6.1%) (38). Most diabetic patients have poor health literacy (mean score: 8.2 out of 20). In Ilam, Iran, only 27.2% of diabetic patients had adequate health literacy. The results of both these studies agree with the present findings (21, 39).

However, the present findings disagree with the results obtained in Sweden, in which more than half of diabetic patients had adequate functional health literacy (40). Better education in participants from developed countries has led to this difference since education is one of the factors associated with health literacy. Inadequate health literacy appears to be an issue in diabetic patients in many different societies, and the reason for this similar pattern in different societies is not clear. Studies have shown, however, that health literacy in diabetic patients has relationships with self-confidence, self-management behavior, glycemic control and health outcomes (41), which shows the need for greater attention to health literacy in diabetic patients.

The present findings showed that the mean health literacy score was higher in diabetic men (50.06) compared to diabetic women (47.97) although the difference was not significant; in other words, men have higher levels of health literacy. Not many studies have yet been conducted on the differences in the health literacy of diabetic patients in terms of gender, but the few studies conducted on this subject have produced similar results to the present study. In a study conducted in Pakistan (36) to compare health literacy between women and men, although the difference was not significant, the ratio of women with inadequate health literacy in the women’s group was greater than the ratio of men with inadequate health literacy in the men’s group, which agrees with the present findings. The health literacy score was higher in men (58.6±21.22) than in women (54.8±21.3), and the ratio of men with adequate health literacy in the men’s group was greater than the ratio of women with adequate health literacy in the women’s group, which agrees with the present findings, even though these differences were not statistically significant (37).

Although the differences were not significant, the ratio of men with high health literacy was greater than the ratio of women with high health literacy, which concurs with the present findings (16). Although the reason for these differences is not clear, it seems that, in many societies, the mean score of health literacy or the prevalence of adequate health literacy levels are higher in men than in women. Some reasons could be the higher level of education in men compared to women and the observation that men have more information as a result of the various jobs at which they work and the resultant extensive social communication. The precise reasons causing this difference require further studies.

In the present study, the PRISMA guidelines were used for the research design and the systematic review steps. To the researchers’ knowledge, this study is the first systematic review and meta-analysis of health literacy in diabetic patients in Iran.

This analysis examined cross-sectional studies and therefore could not assess the causal relationship between health literacy and diabetes. The majority of the studies entered into the meta-analysis had not investigated the confounding factors affecting health literacy levels, and their results may, therefore, be biased. There could be some selection bias in some of the studies entered into the meta-analysis due to certain confounding factors or factors affecting health literacy not investigated in those studies, although the overall selection bias and its effect on the results of the meta-analysis cannot be accurately assessed. The dissimilar questionnaires used in the reviewed studies was another limitation of this study due to different scoring system in the studies. Although attempts were made to resolve this issue by recalculating the scores obtained from the different questionnaires (range:0–100), the details examined in each questionnaire were unique, and this limitation should be considered in the interpretation of the results.

## Conclusion

Health literacy is not high in Iranian diabetic patients and is rather inadequate in most of them. Moreover, diabetic women have lower levels of health literacy compared to diabetic men. Due to the importance of health literacy in the control of diabetes and its outcomes, it is necessary for the health authorities to take effective measures to improve the health literacy of diabetic patients. Policymakers and practitioners in the healthcare system must try on improving health literacy among patients with diabetes. As a suggestion, effective diabetic patient education and engagement will require some simplification, and diabetic patients need more convenient and meaningful access to their health information and to resources that will help them to make informed care decisions.

## Ethical considerations

Ethical issues (Including plagiarism, informed consent, misconduct, data fabrication and/or falsification, double publication and/or submission, redundancy, etc.) have been completely observed by the authors.
